# A Quick Method for the Determination of the Fraction of Freebase Nicotine in Electronic Cigarettes

**DOI:** 10.1021/acs.chemrestox.2c00371

**Published:** 2023-07-05

**Authors:** Amira Yassine, Cynthia Antossian, Rachel El-Hage, Najat A. Saliba

**Affiliations:** † Department of Chemistry, Faculty of Arts and Sciences, American University of Beirut, Riad El Solh, Beirut 1107 2020, Lebanon; ‡ Center for the Study of Tobacco Products, Virginia Commonwealth University, 100 W. Franklin St. Suite 200, Richmond, Virginia 23220, United States

## Abstract

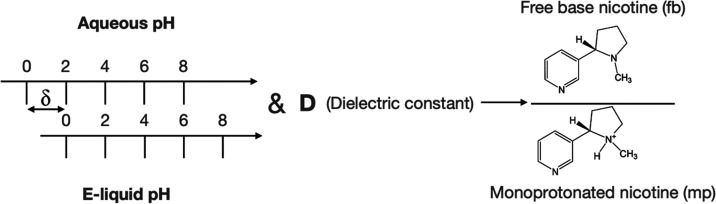

Recently, many electronic cigarettes (ECIGs) manufacturers have begun offering e-liquids, known as “nicotine salts”. These salts that have started gaining big popularity among users can be formed by adding weak acid to e-liquid mixtures consisting of propylene glycol (PG), vegetable glycerin (VG), flavors, and nicotine. The latter can exist in two forms: monoprotonated (mp) and freebase (fb) based on the pH of the matrix. Over the years, the determination of the fraction of fb was found important to policymakers as the prevalence of this form in ECIGs has been associated with the harshness sensory of inhalable aerosols. Liquid–liquid extraction (LLE), ^1^H NMR, and Henderson–Hasselback have been developed to deduce the fraction of fb; however, these methods were found to be time-consuming and have shown some challenges mainly due to the presence of a non-aqueous matrix consisting of PG and VG. This paper presents a quick non-aqueous pH measurement-based method that allows a quick determination of the fraction fb by just measuring the pH and the dielectric constant of the e-liquid. Then, by inputting these values into an established mathematical relationship, the fraction fb can be deduced. The relationship between pH, dielectric constant, and fb relies on knowing the values of the acidity dissociation constants of nicotine, which were determined for the first time in various PG/VG mixtures using a non-aqueous potentiometric titration. To validate the proposed method, the fraction fb was determined for commercials and lab-made nicotine salts utilizing the pH and LLE methods. The variation between the two methods was (<8.0%) for commercial e-liquids and lab-made nicotine salts containing lactic acid and salicylic acid. A larger discrepancy of up to 22% was observed for lab-made nicotine salts containing benzoic acid, which can be attributed to the stronger affinity of benzoic acid to toluene in the LLE method.

## Introduction

### Nicotine Salts and Inhalation Harshness

E-cigarettes (ECIGs) are battery-powered devices introduced to the market as an alternative to combustible cigarettes.^1−4^ Since their release, they gained big popularity that continues to rise to reach an epidemic level, especially among youth.^5−7^ Even though the main operating principle remained the same, ECIGs evolved remarkably in shape, design, and composition to make devices more appealing to users and more effective to deliver nicotine.^8^ The ECIGs line of evolution is usually drawn from cartridge-based to tank-based, which allows users to customize their device features like liquid nicotine concentration and device power.^9−11^

**Figure 1:**
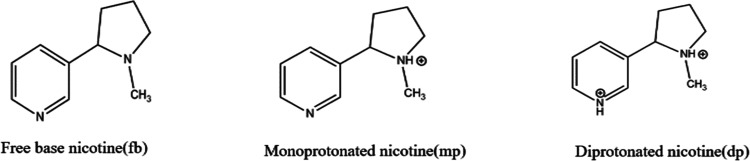
Nicotine forms.

In 2015, JUUL manufacturers introduced cartridge-based ECIGs, which succeeded to deliver nicotine efficiently as it relies on aerosolizing a liquid containing a high concentration of nicotine salts.^12^ These salts can be obtained by the addition of an acid to the mixture consisting mainly of propylene glycol (PG), vegetable glycerin (VG), flavors, and nicotine. The latter is an alkaloid with a weak basic nature, which has two basic nitrogen groups in its chemical structure. Therefore, it can exist in three forms: freebase (fb), monoprotonated (mp), and diprotonated (dp) nicotine depending on the pH of the matrix as shown in Figure 1.

These forms especially fb and mp exhibited different chemical^13^ and physiological behaviors.^14^ Studies have shown that inhalation of nicotine in the fb form is responsible for a harshness perception,^15,16^ whereas the other forms did not induce the same perception.^17^ Hence, the determination of the fb fraction was found important to policymakers as a low fraction of fb will lead to less harshness in the throat and thus makes vaping more likely to be appealing for vapers.^18^

### Methods for the Determination of the Fraction of Free Base Nicotine in E-Liquids

At present, there are different proposed methods for the determination of nicotine forms in nicotine salt-based e-liquid. El Hellani et al. developed an extraction method that used a solvent system consisting of water and toluene and exploits the fact that, due to its chemical structure, toluene will extract only fb from a solution containing both fb and mp.^19^ This liquid–liquid extraction (LLE) was able to give an accurate value of the fb, but it is like any LLE, requiring many extraction steps, a large amount of solvent, and a long time to be completed.^20^ Duell et al. used a ^1^H NMR spectroscopy to deduce the fraction of fb in e-liquid without doing any dilution.^21^ It was reported that this detection would not be easy all the time. In fact, if the e-liquid has acid in high concentrations, this can lead to the presence of nicotine in the dp form, which will contribute meaningfully to the ^1^H NMR spectrum making the detection challenging.^22^ Likewise, in some cases, the presence of one or many flavors that have peaks in the nicotine spectral regions can induce errors in the detection.^22^ The Henderson–Hasselbach method was also proposed and widely used for this determination.^19,22−34^ It consists of a dilution of the e-liquid (1:10) with water, and then, a pH measurement is carried out using a glass electrode calibrated by an aqueous buffer solution. Thus, it can give only the fb of the e-liquid diluted in water.

The three reviewed methods are time-consuming, expensive, and require the help of a trained chemist to perform them. They also have encountered some limitations due to the presence of a non-aqueous matrix consisting of PG and VG. So, finding a quick method that allows the determination of fb by researchers who are not necessarily chemists is crucial. This paper presents for the first time a non-aqueous pH measurement method that allows a quick determination of fb without any dilution with water. This method consists of determining two parameters, mainly the pH and the dielectric constant of the e-liquid, plugging them into the established association presented here for the first time, and then computing the fb fraction. Note that the relationship between pH, dielectric constant, and fb relies on knowing the values of the acidity dissociation constants of nicotine, which were also determined for the first time in different PG/VG mixtures using a non-aqueous potentiometric titration. Since there are no available p*K*_a_ values for nicotine in PG/VG mixtures, and there are only a few p*K*_a_ values for benzoic acid in these mixtures, we also determined the p*K*_a_ of benzoic acid to prove the validity of the non-aqueous potentiometric method used.

### Equation Formulation

The calculation of the nicotine base and its conjugated acid in an ECIG liquid mixture will require the determination of many physical and chemical parameters as described below.

### Equilibrium Constants of Acid Dissociation Reactions

#### Conjugate Acid of a Base

As a general definition, when a conjugate acid of a base BH^+^ (i.e., protonated nicotine) is dissolved in a certain solvent S (i.e., PG, VG, water, etc.), the equilibrium shown in eq 1 occurs
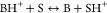
1

The equilibrium constant *K*_c_ is equal to
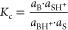
2where SH^+^ is the protonated form of the solvent and *a* is the activity of a certain species with *a* being equal to
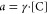
3[C] is the concentration of the specie,^35^ and γ is the activity coefficient. It can be calculated for any ion of charge *z* using the Davis equation^13,35,36^
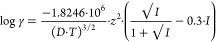
4whereas in eq 4, *I*, the ionic strength, represents the summation of all anions and cations in solution, and *c*_i_ is the concentration of the ion with
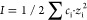
5where *D* is the dielectric constant of the solvent or mixture of solvents and *T* is the temperature.

For a dilute solution, [S] is constant, and the activity coefficients for neutral species remain close to unity.

The acidity dissociation constant (*K*_a_) of eq 1 is then defined as
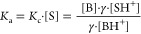
6Assuming that all the H^+^ ions are bound to the solvent molecules, [SH^+^] can be equivalent to [H^+^] so
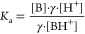
7

Based on eq 4, *K*_a_ depends on the concentrations of the base and the conjugated acid and the activity coefficient γ. Azzouz et al.^37^ showed that there is a linear relationship between p*K*_a_ and the dielectric constant (*D*) of the solvent with
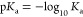
8and
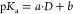
9so calculating p*K*_a_ as a function of *D* will help determine the ratio of base and conjugated acid using eq 7.

#### Neutral Acid

Using the same analogy and calculation used in the case of a conjugate acid of the base (details in Supporting Information-Part 1), we can deduce that
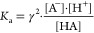
10Also, similar to the conjugate acid of a base, the relationship between p*K*_a_ and *D* can be calculated based on eq 11.
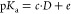
11

## Experimental Procedure

### Apparatus

Potential measurements were carried out using a Mettler Toledo pH meter equipped with an Inlab viscous electrode specific for a viscous non-aqueous medium. The electrode was stored in a KCl storage solution and calibrated using standard aqueous buffer solutions. Measured pH is considered as pH^app^.
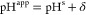
12where pH^s^ is the real pH of the solution.^35^

The correction, δ, is specific to each solvent
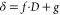
13

It can be determined by using a buffer solution of known p*K*_a_ in a given solvent or by titrating a strong acid in a solvent S by a strong base in the same solvent S.^35^

### Chemicals and Reagents

PG (99.5%; CAS no. 57-55-6), VG (99–101%; CAS no. 56-81-5), and hydrochloric acid (HCl; 37%; CAS no. 7647-01-0), standardized tetrabutylammonium hydroxide in methanol solution (*C* = 1 M), salicylic acid, and benzoic acid crystals were purchased from Sigma-Aldrich. Pure nicotine (CAS registry number 54-11-5) was purchased from Acros Organics and lactic acid from Fluka Analytical.

### Reagent Preparation

Hydrochloric acid (HCl), tetrabutylammonium hydroxide (ROH), and nicotine solutions were prepared in water and in different % volume/volume (% v/v) of PG/VG (100/0, 50/50, 0/100). These solutions of similar concentration were prepared (*C* = 1 ×10^–3^ M) by pipetting the required volume, diluting it in the specific solvent, and then sonicating the mixture for 5 h before use.

Benzoic acid solutions were prepared in water and in different % volume/volume (% v/v) of PG/VG (100/0, 50/50, 0/100). These solutions were prepared at the same concentration (*C* = 1 ×10^–3^ M) by measuring the required mass and then diluting it in the specific solvent. Then, the prepared solutions were sonicated for 5 h before use.

### Determination of δ

The correction, δ, is specific to each solvent and was determined by using the two-step procedure described below.^35^

#### Step 1: Standardization of HCl Prepared in a Solvent S

20 mL of the prepared HCl solution in a solvent S was pipetted into a beaker. The transferred solution was titrated by the standardized strong base of tetrabutylammonium hydroxide solution (ROH), which was prepared in methanol (*C* = 1 M) using pH measurement for every 5 μL added. The pH was then plotted versus the volume of the titrant. Using the derivative method, *C*_0_ was deduced (calculations are detailed in Supporting Information-Part 2.1).

#### Step 2: Titration of HCl Prepared in a Solvent S by ROH Prepared in the Solvent S

20 mL of the prepared standardized HCl solution in a solvent S was pipetted into a beaker. HCl was then titrated by the diluted ROH by measuring the pH of the solution for every 2 mL addition. Finally, pH^s^ was calculated and δ was deduced (calculations are detailed in Supporting Information-Part 2.2).

### Determination of the p*K*_a_ of Nicotine

The p*K*_a_ of nicotine was determined following the two-step procedure described below.

#### Step 1: Standardization of HCl Prepared in the Solvent S

Standardization was done using the same procedure described above.

#### Step 2: Titration of Nicotine Prepared in the Solvent S by the Standardized HCl Prepared in the Solvent S

20 mL of the prepared nicotine solution in solvent S was pipetted into a beaker and then titrated by the standardized HCl solution using a pH titration method. The pH was plotted versus the volume of the titrant added (an example is shown in Supporting Information-Part 3.1). The derivative method was then used to determine *V*_eq_. Finally, the p*K*_a_ was deduced easily from the half equivalence point (details about calculations can be found in Supporting Information-Part 3.2). This procedure was first applied to solutions in water and then applied to different % v/v of PG/VG (100/0, 50/50, 0/100).

### Determination of the p*K*_a_ of Benzoic Acid

The determination of the p*K*_a_ of benzoic acid was done using the three-step procedure described below.

#### Step 1: Standardization of HCl Prepared in the Solvent S

Standardization was done using the same procedure described above.

#### Step 2: Standardization of ROH Prepared in the Solvent S

Standardization was done using the same procedure described above.

#### Step 3: Titration of Benzoic Acid

20 mL of benzoic acid that was prepared in the solvent S was pipetted into a beaker. Using a pH titration method, the prepared solution was titrated by the standardized ROH solution, and the pH was plotted versus the volume of the titrant added (an example is shown in Supporting Information-Part 4.1). The derivative method was then used to determine Veq. Finally, the p*K*_a_ was deduced easily from the half equivalence point (details about calculations can be found in Supporting Information-Part 4.2). This procedure was first applied to solutions in water and then was applied to different % v/v of PG/VG (100/0, 50/50, 0/100).

### pH and *D* Measurement to Determine the Fraction *fb*

As detailed in Supporting Information-Part 5
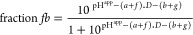
14

By determining the constant *a*, *f*, *b*, and *g* using the experimental procedure described above, eq 14 will remain with only two unknowns: pH^app^ (depends on the composition of the liquid and type of acid) and the dielectric constant (depends on the type of the solvents). By using this equation, we can therefore determine the fraction fb in any e-liquid mixture (regardless of the type of acids and solvents present) by just measuring the pH^app^ using a non-aqueous pH meter and the dielectric constant using a dielectric constant meter. Alternatively, the % volume of PG and VG can be determined by adopting a previously described method,^38^ and then, the dielectric constant can be calculated using the formula^39^
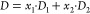
15where *D*_1_, the dielectric constant of PG, is 27.5^40^ at 25°C, *D*_2_, the dielectric constant of VG, is 42.5^41^ at 25 °C, and *x*_1_ and *x*_2_ are the mole fraction of PG and VG, respectively (details about the calculation of mole fractions from % volume can be found in Supporting Information-Part 6).

### Method Validation

Four flavored N-JOY e-liquids of different nicotine lactate concentrations (28 and 58 mg/L) and 18 lab-made nicotine benzoate, nicotine salicylate, and nicotine lactate e-liquids (molar ratio acid/nicotine 1:1) were prepared at two different nicotine concentrations (12 and 60 mg/L), and in various PG/VG ratios (100/0, 70/30, 30/70) (details about the preparation of lab-made nicotine salts can be found in Supporting Information-Part 7). Fraction fb was calculated after measuring the pH and using the LLE^19^ method. The pH of each liquid was determined using a Mettler Toledo pH meter equipped with an Inlab viscous electrode. The % volume of PG/VG was determined using the previously described method.^38^ The dielectric constant of each e-liquid was calculated using eq 15.

## Results

The real pH of the solutions (pH^s^) in water and % v/v of PG/VG: 100/0, 50/50, 0/100 depends on the δ values, which are calculated using eq 12 (Table 1). The respective dielectric constants of water, 100/0 PG/VG and 0/100 PG/VG are reported to be 78.5,^42^ 27.5,^40^ and 42.5,^41^ whereas the dielectric constant of the 50/50 PG/VG solution was calculated using eq 15.

**Table 1:** Experimental Values of δ, p*K*_a_ Benzoic Acid, and p*K*_a_ Nicotine in Different Solvents

	δ	p*K*_a_ benzoic acid	p*K*_a_ nicotine
100/0 PG/VG	–2.05	8.83	8.79
50/50 PG/VG	–1.78	8.11	8.63
0/100 PG/VG	–1.59	7.01	8.50
100% water	0	4.20	8.02

The plot of the δ values as a function of *D* proved the linear relationship δ = *f*·*D* + *g*(*R*^2^ = 0.995) with *f* = 0.04 and *g* = −3.22, meaning that for a known *D*, the δ value can be calculated using the newly established relationship and the real pH can be deduced using eq 12.

The p*K*_a_^s^ of nicotine and benzoic acid in water and different % v/v of PG/VG: 100/0, 50/50, 0/100 were also determined. Results showed that the determined values of p*K*_a_ of nicotine in water of 8.01,^43^ p*K*_a_ of benzoic acid in water of 4.20,^44^ and 100/0 PG/VG of 8.83^45^ were consistent with the reported values in the literature, which proved the validity of the potentiometric method that was used. The p*K*_a_ of nicotine in PG/VG (100/0, 50/50, and 0/100) and those of benzoic acid in PG/VG (50/50 and 0/100) were determined in this study for the first time (Table 1). Moreover, by plotting the p*K*_a_ values versus the dielectric constant of the mixture for nicotine (*y* = −0.015*x* + 9.19, *R*^2^ = 0.982) and benzoic acid (*y* = −0.090*x* + 11.14, *R*^2^ = 0.988), the constants *a*, *b*, *c*, and *e* for eqs 9 and 11 were deduced to be −0.015, 9.19, −0.090, and 11.14, respectively. These equations can be used to find the pKa of nicotine and benzoic acid for any mixture with a known dielectric constant.

Determining the constants, *a*, *b*, *c*, *e*, *f*, and *g*, has allowed the computation of the eq 16 (final form of eq 14), which can be used to determine the fraction *fb* in any e-liquid after appropriate measurements of pH and *D* are made.
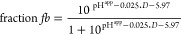
16

For the validation, fractions *fb* and *mp* were calculated from experimental values using eqs 16 and 17, respectively (details are found in Supporting Information-Part 8). pH, % volume PG/VG, calculated dielectric constant, and [mp] were summarized in Table 2.
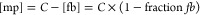
17

**Table 2:** Experimental Values of the Fraction *fb*, % Volume PG/VG, pH of the Tested E-Liquids, Fraction *fb*, and [mp] Determined Using pH and LLE and % Difference of *fb* Fraction Using the pH Method and the LLE

e-liquid	nicotine concentration (mg/mL)	% volume PG	% volume VG	D	pH	fraction fb using pH method	fraction fb using LLE	[mp]_pH_	[mp]_LLE_	% difference
NJOY Ace-Classic Tobacco	28	52.33	47.67	34.67	6.06	0.143	0.150	0.857	0.850	0.8
NJOY Ace-Classic Tobacco	58	45.49	54.51	35.69	6.16	0.166	0.090	0.834	0.910	8.4
NJOY Ace-Menthol	28	47.99	52.01	35.32	6.02	0.128	0.121	0.872	0.879	0.8
NJOY Ace-Menthol	58	46.01	53.99	35.62	6.16	0.166	0.103	0.834	0.897	7.0
nicotine lactate	12	100	0	27.50	6.06	0.202	0.178	9.580	9.860	2.8
nicotine lactate	60	100	0	27.50	6.28	0.295	0.244	42.275	45.380	6.8
nicotine lactate	12	70	30	32.02	6.29	0.249	0.237	9.017	9.153	1.5
nicotine lactate	60	70	30	32.02	6.44	0.318	0.268	40.894	43.906	6.9
nicotine lactate	12	30	70	38.02	6.53	0.289	0.293	8.529	8.483	0.5
nicotine lactate	60	30	70	38.02	6.54	0.294	0.244	42.361	45.342	6.6
nicotine salicylate	12	100	0	27.50	5.42	0.055	0.039	11.343	11.535	1.7
nicotine salicylate	60	100	0	27.50	5.22	0.035	0.009	57.886	59.462	2.7
nicotine salicylate	12	70	30	32.02	5.46	0.047	0.031	11.440	11.634	1.7
nicotine salicylate	60	70	30	32.02	5.71	0.080	0.041	55.198	57.525	4.0
nicotine salicylate	12	30	70	38.02	5.58	0.044	0.048	11.476	11.423	0.5
nicotine salicylate	60	30	70	38.02	5.79	0.069	0.046	55.864	57.233	2.4
nicotine benzoate	12	100	0	27.50	6.07	0.205	0.054	9.535	11.352	16.0
nicotine benzoate	60	100	0	27.50	6.22	0.267	0.050	43.950	56.982	22.9
nicotine benzoate	12	70	30	32.02	6.16	0.197	0.070	9.637	11.156	13.6
nicotine benzoate	60	70	30	32.02	6.23	0.224	0.045	46.581	57.306	18.7
nicotine benzoate	12	30	70	38.02	6.09	0.129	0.059	10.455	11.293	7.4
nicotine benzoate	60	30	70	38.02	6.14	0.142	0.070	51.468	55.824	7.8

For the LLE method, the fractions *fb* and [mp] were determined as described in previous work,^19^ and the % difference between the two methods was summarized the results in Table 2. These results showed that the difference between the two methods calculated ranged for 4 commercial e-liquids between 0.8 and 8.3% and for 18 lab-made nicotine salts containing lactic acid between 0.5 and 6.9%, salicylic acid between 0.5 and 4.0%, and benzoic acid between 7.4 and 22.9%.

## Discussion

The primary aim of this study was to develop a quick non-aqueous pH measurement-based method that allows the determination of the fraction fb without any dilution with water. This is done by measuring the pH^app^ using a non-aqueous pH meter and the dielectric constant using a dielectric constant meter (or by calculating the dielectric constant after determining the % volume PG/VG) and inputting these values into eq 16. The p*K*_a_ of nicotine and p*K*_a_ of benzoic acid were determined using a potentiometric titration in water and different % v/v PG/VG (100/0, 50/50, and 0/100). Results showed that the p*K*_a_ of nicotine in different PG/VG ratios cannot be assumed to be equal to the p*K*_a_ of nicotine in water. In fact, the % difference between p*K*_a_ of nicotine in water and 100% PG was as high as 77.0%, which proves that the usage of the Henderson–Hasselbach method is not valid for the determination of the fraction of *fb*. The effect of the dilution in water was previously confirmed by Pankow et al., who showed that the analytical error of presuming that the fraction *fb* in water after 1:10 dilution is equal to the fraction *fb* of an e-liquid is large.^46^

The fraction *fb* determined through the pH method presented in this study exhibited consistency with the values previously reported by Duell et al., using ^1^H NMR.^47^ When preparing nicotine benzoate salt in 32/68 PG/VG with a nicotine concentration of 54 mg/mL, a *fb* fraction of 0.14 was obtained.^47^ This value differs by 1.4% from the *fb* fraction calculated in our study for nicotine benzoate in a 30/70 PG/VG nicotine benzoate with a nicotine concentration of 60 mg/mL (Table 2).

Comparing the results to the LLE methods, it was found that the fractions *fb* and [mp] for commercial e-liquids with a nicotine concentration of 28 mg/mL were both 0.8% for NJOY Ace-Menthol and NJOY Ace-Classic Tobacco. Similarly, a low percentage difference (<5%) was calculated for the lab-prepared nicotine salt with a concentration of 12 mg/mL in salicylic and lactic acids and in various PG/VG ratios (Table 2). However, nicotine benzoate at a nicotine concentration of 12 mg/mL in different PG/VG ratios had a higher percentage difference ranging from 7.4 to 16.0%.

Commercial e-liquids containing nicotine at a concentration of 58 mg/mL as well as lab-made nicotine lactate and salicylate with a nicotine concentration of 60 mg/mL (in different PG/VG ratios) exhibited a % difference (<8.3%). However, the difference for lab-made nicotine benzoate ranged from 7.8 to 22.9%.

The significant difference of up to 22% between the pH and LLE methods when benzoic acid was utilized can be attributed to the limited selectivity of toluene in LLE process. Previous studies have indicated that toluene has a tendency to extract acids, particularly benzoic acid, which was identified in the extracted samples.^48^ Additionally, toluene has been found to extract flavors and nicotine.^46,48^ Consequently, this extraction process can lead to a shift in the fb fraction values, thereby elucidating the larger discrepancies observed between the two methods.

In conclusion, this study presents a simplified method for determining the fb fraction in an e-liquid without the need for dilution or complex experimental procedure such as LLE and ^1^H NMR. The method involves measuring only the dielectric constant and pH of the solution. Furthermore, this approach enables the calculation of new variables such as pH^app^, δ, *D*, p*K*_a_s of nicotine and benzoic acid in non-aqueous PG/VG media. The identified correlations between the p*K*_a_ values of nicotine and benzoic acid and the dielectric constants of the solvent can be considered as a novel means to estimate the pKa of these compounds in any mixture with known dielectric constants.

## Supporting Information Available

Additional experimental details and methods, including the titration curves of nicotine and benzoic acid (PDF)

## Abbreviations

ECIGs: electronic cigarettes
fb: free base nicotine
mp: monoprotonated nicotine
dp: deprotonated nicotine
PG: propylene glycol
VG: vegetable glycerin
LLE: liquid–liquid extraction
