# Supplementary material for: A Quick Method for the Determination of the Fraction of Freebase Nicotine in Electronic Cigarettes
Source: Chem Res Toxicol. 2023 Jul 5;36(7):1021–7. doi: 10.1021/acs.chemrestox.2c00371 (PMC10354800; doi:10.1021/acs.chemrestox.2c00371)
Supplement: Supplementary file 1 — tx2c00371_si_001.pdf [file tx2c00371_si_001.pdf]

SUPPORTING INFORMATION

Additional experimental details, and methods, including the titration curves of nicotine and benzoic acid.

A quick method for the determination of the fraction of freebase nicotine in electronic cigarettes

Amira Yassine,[†] Cynthia Antossian, † Rachel El-Hage,[†,§] , and Najat A. Saliba.[†,§,]

† Department of Chemistry, Faculty of Arts and Sciences, American University of Beirut, Riad El Solh, Beirut 1107 2020, Lebanon.

§ Center for the Study of Tobacco Products, Virginia Commonwealth University, 100 W. Franklin St. Suite 200, Richmond, VA 23220, United States.

Correspondence to: Najat A.Saliba, Tel: +961 1 350000/3992. E-mail: ns30@aub.edu.lb.

Table of contents

1. **Equilibrium constants of acid dissociation reactions (neutral acid).**
2. **Determination of δ.**
3. **Titration of nicotine.**
4. **Titration of benzoic acid.**
5. **Relationship between fraction fb, pH^{app}, and D.**
6. **Determination of the dielectric constant.**
7. **Lab-made e-liquids preparation.**
8. **Determination of the [mp].**

---

1. Equilibrium constants of acid dissociation reactions (neutral acid)

When a neutral acid (i.e., benzoic acid) is dissolved in a certain solvent S (for example PG, VG, water, etc.…), the equilibrium shown in Equation S_1 occurs

$$HA + S \leftrightarrow A^- + SH^+ \qquad \text{(Equation } S_1\text{)}$$

The equilibrium constant of Equation S_1 is

$$K_c = \frac{a_{A^-} . a_{SH^+}}{a_{HA} . a_S} \qquad \text{(Equation } S_2\text{)}$$

With

$$K_c . [S] = \frac{\gamma . [A^-] . \gamma . [SH^+]}{[HA]} \qquad \text{(Equation } S_3\text{)}$$

The acidity dissociation constant is then

$$K_a = K_c . [S] = \frac{\gamma . [A^-] . \gamma . [SH^+]}{[HA]} \qquad \text{(Equation } S_4\text{)}$$

Ka becomes then equal to

$$Ka = \gamma^2 . \frac{[A^-] . [H^+]}{[HA]} \qquad \text{(Equation } S_5\text{)}$$

2. Determination of δ

2.1. Standardization of HCl in a solvent S: Calculations

The titration reaction is $HCl + ROH \rightarrow RCl + H_2O$ (Equation S_6)

If HCl (C_0 ,V_0) is titrated with a standardized ROH (1 M, V_1).

At equivalence:

$n\ H^+ = n\ OH^-$ thus $C_0 . V_0 = 1 . V_{eq}$ where V_{eq} can be found using the derivative method. It consists of plotting $\frac{dpH}{dV}$ function of the volume of ROH added. To calculate $\frac{dpH}{dV}$ the following calculation must be used:

For two successive measurements in a pH vs. Volume plot: (V_1, pH_1) and (V_2, pH_2), the derivative is: $\frac{dpH}{dV} = \frac{pH_2 - pH_1}{V_2 - V_1}$. V_{eq} corresponds to the highest peak.

Then, we can deduce C_0.

2.2. Titration of HCl in a solvent S by ROH in a solvent S: Calculations

2.2.1. Relation between δ and $[H^+]^s$

pH-scales in aqueous and non-aqueous media can be defined by:

pH^S = -log $a\ H^+$ (Equation S_7)

The hydrogen-ion activity can be defined by:

$a\ H^+ = \gamma\ .\ [H^+]^s$ (Equation S_8)

in which γ is the activity coefficient and $[H^+]^s$ is the real concentration of $[H^+]$ in the solution.[1]

By combining Equation S_7 and Equation S_8, the below equation is obtained:

$pH^S = -\log \gamma\ .\ [H^+]^s$ (Equation S_9)

Thus, $pH^S = -\log \gamma - \log[H^+]^s$ (Equation S_{10})

$pH^{app} = pH^s + \delta$ (Equation S_{11})

Thus, $pH^S = pH^{app} - \delta$ (Equation S_{12})

By using Equation S_{10} and Equation S_{12}, the following equation can be obtained:

$pH^S = pH^{app} - \delta = -\log\gamma - \log[H^+]^s$ (Equation S_{13})

$\delta = pH^{app} + \log\gamma + \log[H^+]^s$ (Equation S_{14})

Based on Equation S_{14}, to find δ a pH measurement must be done to find pH^{app}, and calculations must be done to deduce γ and $[H^+]^s$.

2.2.2. Determination of $[H^+]^s$

The titration reaction is $HCl + ROH \rightarrow RCl + H_2O$ (Equation S_{15})

If HCl (C_0, V_0) is titrated with a standardized ROH (C_1, V_1).

2.2.2.1. At equivalence:

$n\ H^+ = n\ OH^-$ thus $C_0 . V_0 = C_1 . V_{eq}$.

V_{eq} can be found using the derivative method.

2.2.2.2. Before the equivalence point:

The acid is in excess, thus, the composition after adding V_1 volume of ROH will be as shown in Table S_1.

Table S_1 : Titration of HCl with ROH.

	HCl +	ROH →	RCl +	H_2O
t_0	$C_0 . V_0$	$C_1 . V_1$	-	-
t_f	$C_0 . V_0 - C_1 . V_1$	-	$C_1 . V_1$	$C_1 . V_1$

Thus, $[H^+]^S = \frac{C_0 . V_0 - C_1 . V_1}{V_0 + V_1}$. This formula can be used for the determination of $[H^+]^s$ at each point before the equivalence.

2.2.3. Determination of the activity coefficient

The activity coefficient γ of an ion of charge z can be calculated using the Davis equation:

$$\log\gamma = -\frac{1.8246\ .\ 10^6}{(D\ .\ T)^{3/2}}\ .z^2.(\frac{\sqrt{I}}{1+\sqrt{I}} - 0.3\ .\ I) \qquad \text{(Equation S}_{16}\text{)}^{1-3}$$

For the titration of HCl by ROH, the ionic strength can be found as explained below.

<u>2.2.3.1. At V=0 ml:</u>

We have in the solution: H^+ and Cl^-.

$[H^+] = [\ Cl^-] = C_0$.

Thus, $I = 1/2 \sum ci\ .zi^2 = \frac{1}{2}\ .([H^+]+[\ Cl^-]) = \frac{1}{2}\ .([H^+]+[H^+]) = [H^+] = C_0$.

<u>2.2.3.2. At V< V eq:</u>

We have in the solution after adding V of ROH: H^+, R^+, $and\ Cl^-$.

$[R^+] = \frac{C_1\ .\ V_1}{V_0+V_1}$, $[\ Cl^-] = \frac{C_0\ .\ V_0}{V_0+V_1}$, $[H^+] = \frac{C_0\ .\ V_0 - C_1\ .\ V_1}{V_0+V_1}$.

$$I = \frac{1}{2}\ .([H^+]+[\ Cl^-] + [R^+]) = \frac{1}{2}\ .[\frac{C_0\ .\ V_0 - C_1\ .V_1}{V_0+V_1} + \frac{C_1\ .\ V_1}{V_0+V_1} + \frac{C_0\ .\ V_0}{V_0+V_1}] = \frac{1}{2}\ .[\frac{2\ .\ C_0\ .V_0}{V_0+V_1}] = \frac{C_0\ .V_0}{V_0+V_1}.$$

By determining I, the calculation of the activity coefficient will be easy using equation S_{16}.

Now, determination of the value of δ was done using Equation S_{14} after using pH^{app} , and by calculating $\log\gamma$ and $[H^+]^s$ of the points determined before the equivalence. Similar δ values were obtained for all the points.

3. Titration of nicotine

3.1. Titration curve

An example of the titration curve obtained is shown in Figure S_1.

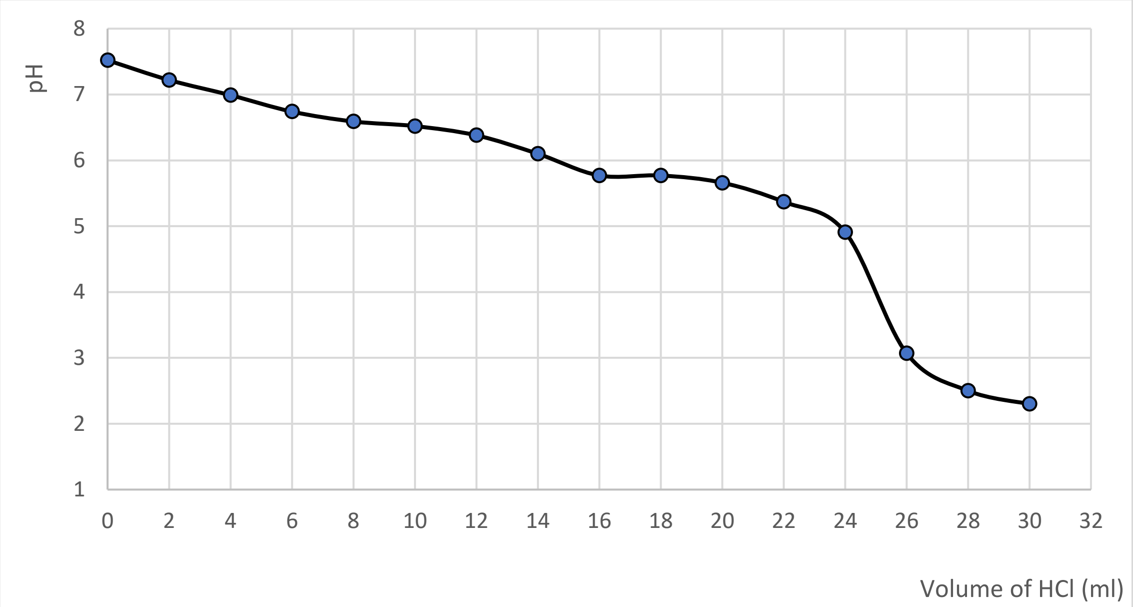

Figure S_1: Titration of nicotine in 100/0 PG/VG by HCl in 100/0 PG/VG.

3.2. Calculations

$pH^s = pKa + \log \frac{[B]}{[BH+]}$ (Equation S_{17})[1]

Using Equation S_{12} and Equation S_{17}:

$pH^{app} - \delta = pKa + \log \frac{[B]}{[BH+]}$ (Equation S_{18})

$pH^{app} = pKa + \log \frac{[B]}{[BH+]} + \delta$ (Equation S_{19})

At half equivalence, $[B] = [BH^+]$ so at this point:

$pH^{app} = pKa + \delta$ (Equation S_{20})

δ is already determined and pH^{app} can be found from the curve and thus pKa can be deduce easily.

4. Titration of benzoic acid

4.1. Titration curve

An example of the titration curve obtained is shown in Figure S_2.

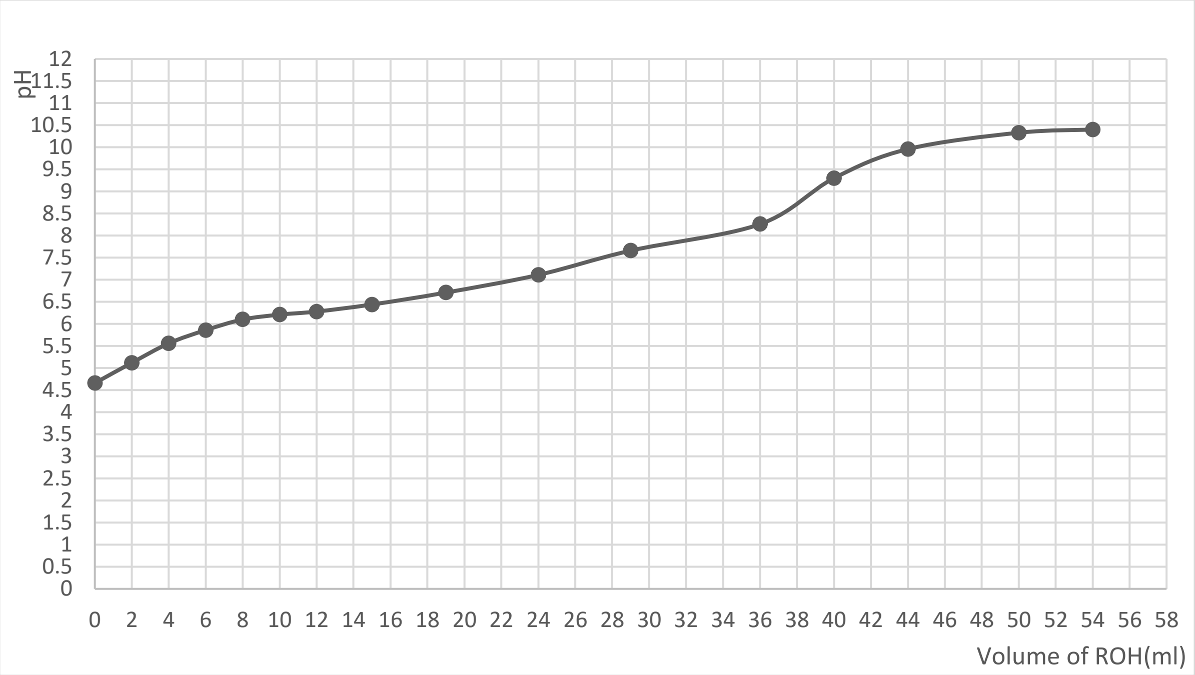

Figure S_2: Titration of benzoic acid in 100/0 PG/VG by tetrabutylammonium hydroxide in 100/0 PG/VG.

4.2. Calculations

$K_a = \frac{\gamma\,.[A^-]\,.\,\gamma\,.[H^+]}{[HA]}$ (Equation S_4)

By combining Equation S_4 and Equation S_8:

$Ka = \frac{\gamma\,.[A^-]\,.\,aH^+}{[HA]}$ (Equation S_{21})

$\log ka = \log\gamma + \log[A^-] + \log aH^+ - \log\ [HA]$ (Equation S_{22})

$-pKa = \log\gamma + \log\frac{[A^-]}{[HA]} - pH^S$ (Equation S_{23})

$pH^S = pKa + \log\gamma + \log\frac{[A^-]}{[HA]}$ (Equation S_{24}) [1]

By combining Equation S_{12} and Equation S_{24}:

$pH^{app} - \delta = pKa + \log\gamma + \log\frac{[A^-]}{[HA]}$ (Equation S_{25})

Therefore, $pH^{app} = pKa + \log \gamma + \log \frac{[A^-]}{[HA]} + \delta$ (Equation S26)

At half equivalence, $[HA]=[A^-]$ so at this point:

$pH^{app} = pKa + \delta + \log \gamma$ (Equation S27)

δ is already determined, pH^{app} can be found from the curve, ROH and benzoic acid were prepared at an exceptionally low concentration so we can guarantee the activity coefficient to be equal to 1. So, pKa can be easily determined using Equation S27.

5. Relationship between fraction fb, pH^{app}, and D

$pH^{app} = pKa + \log \frac{[B]}{[BH+]} + \delta$ (Equation S19)

If we apply this equation to nicotine we will obtain:

$pH^{app} = pKa_{\text{nicotine}} + \log \frac{[fb]}{[mp]} + \delta$ (Equation S28)

We demonstrated in the paper that:

$pKa_{\text{nicotine}} = a.D + b$ (Equation S29)

and,

$\delta = f.\, D + g$ (Equation S30)

Thus, $pH^{app} = a.\, D + b + \log \frac{[fb]}{[mp]} + f\, .\, D + g$ (Equation S31)

$pH^{app} = (a+f).\, D + (b+g) + \log \frac{[fb]}{[mp]}$ (Equation S32)

$\log \frac{[fb]}{[mp]} = pH^{app} - (a+f).\, D - (b+g)$ (Equation S33)

$\frac{[fb]}{[mp]} = 10^{pH^{app} - (a+f).D - (b+g)}$ (Equation S34)

We know that:

[fb]+[mp] = C (Equation S_{35})

So, [mp]=C-[fb] (Equation S_{36})

By combining S_{34} and S_{36}:

$$\frac{[fb]}{C-[fb]} = 10^{pH^{app} - (a+f).D - (b+g)} \quad \text{(Equation } S_{37}\text{)}$$

$$[fb] = (C-[fb]).\, 10^{pH^{app} - (a+f).D - (b+g)} \quad \text{(Equation } S_{38}\text{)}$$

$$[fb](1+10^{pH^{app} - (a+f).D - (b+g)}) = C \,.\, 10^{pH^{app} - (a+f).D - (b+g)} \quad \text{(Equation } S_{39}\text{)}$$

Since, Fraction fb = $\frac{[fb]}{C}$ (Equation S_{40})

Therefore, $Fraction\ fb = \frac{10^{pH^{app} - (a+f).D - (b+g)}}{1+10^{pH^{app} - (a+f).D - (b+g)}}$ (Equation S_{41})

6. Determination of the dielectric constant

To calculate the mole fraction of PG and VG if we have the % volume fraction of PG and VG. An example is given below:

If we have a volume of V: 30% PG +70% VG.

$$\rho = \frac{m}{V} = \frac{M\,.\,n}{V} \quad \text{(Equation } S_{42}\text{)}$$

so, $n = \frac{\rho\,.V}{M}$ (Equation S_{43})

The density of PG and VG are respectively 1.036 and 1.260 g/cm^3.[4, 5]

The molar mass of PG and VG are respectively 76.09 and 92.10 g/cm^3.[4, 5]

The dielectric constant of PG and VG are respectively 27.5[6] and 42.5[7] at 25°C.

Number of mol of PG = $\frac{1.036\ .\ 0.3\ .V}{76.09}$ = 4.0 . 10^{-3}. V mol.

Number of mol of VG = $\frac{1.260\ .\ 0.7\ .V}{92.10}$ = 9.5 . 10^{-3}. V mol.

Total number of mol =13.5 . 10^{-3}. V mol.

Fraction of PG = $\frac{4.0\ .\ 10^{-3}\ .V}{13.5\ .\ 10^{-3}\ .\ V}$ = 0.30.

Fraction of VG = $\frac{9.5\ .\ 10^{-3}\ .V}{13.5\ .\ 10^{-3}\ .\ V}$ = 0.70.

$D= x_1\ .\ D_1+ x_2\ .\ D_2$ (Equation S_{44})[8]

So, D = 0.30 . 27.5+0.70 . 42.5 = 38.

7. Lab-made e-liquids preparation

18 lab-made nicotine benzoate, nicotine salicylate, and nicotine lactate e-liquids (molar ratio acid/nicotine 1:1) were prepared at two different nicotine concentrations (12 and 60 mg/l), and in various PG/VG ratios (100/0, 70/30, 30/70) using the following procedure. Accurate volume and masses of nicotine and acid for each case were mixed in a 10 mL volumetric flask then the volume is completed to the line mark by adding the appropriate PG/VG mixture. The nicotine salt is then vortexed and sonicated overnight. Later, the liquids were transferred to 20 mL vials. Masses and volumes used for the preparation of each nicotine salt are summarized in Table S_2.

Table S_2: Volume and masses of nicotine and acids used for nicotine salts preparation.

Nicotine Salt	Concentration (mg/ml)	Nicotine volume (µL)	Acid
Nicotine benzoate	12	119	0.0904 g
Nicotine salicylate	12	119	0.1022 g
Nicotine lactate	12	119	55 µL
Nicotine benzoate	60	594	0.4518 g
Nicotine salicylate	60	594	0.5112 g
Nicotine lactate	60	594	276 µL

8. Determination of the [mp]

$$\text{fraction fb} = \frac{[\mathbf{fb}]}{[\mathbf{total\ nicotine}]} \qquad \text{(Equation S45)}$$

thus,

[fb] = fraction fb x C (Equation S46)

C = [mp]+[fb] (Equation S47)

therefore,

[mp] = C-[fb] = C x (1- fraction fb) (Equation S48)

REFERENCES

1. Cox, B. G., *Acids and bases: solvent effects on acid-base strength*. Oxford University Press: Oxford, 2013.
2. Pankow, J. F., Calculating compound dependent gas-droplet distributions in aerosols of propylene glycol and glycerol from electronic cigarettes. *J. Aerosol Sci.* **2017,** *107*, 9-13.
3. Murray, J. W. Activity Scales and Activity Corrections 2004. https://www.ocean.washington.edu/courses/oc400/Lecture_Notes/CHPT6.pdf.
4. Duell, A. K.; Pankow, J. F.; Peyton, D. H., Free-base nicotine determination in electronic cigarette liquids by 1H NMR spectroscopy. *Chem. Res. Toxicol.* **2018,** *31* (6), 431-434.
5. Scientific, T. F. Measuring pH of Non-Aqueous and Mixed Samples https://assets.thermofisher.com/TFS-Assets/LSG/Application-Notes/AN-PHNONAQS-E%201014-RevA-WEB.pdf.
6. T, V., Dielectric Spectroscopic Studies of Propylene Glycol/Aniline Mixtures at Temperatures Between 303K to 323K. *Int. j. res. eng. technol.* **2015**, 2015.
7. Honeywell. Dielectric constant table. https://prod-edam.honeywell.com/content/dam/honeywell-edam/pmt/hps/products/pmc/field-instruments/smartline-level-transmitters/smartline-guided-wave-level-transmitters/pmt-hps-dielectric-constant-table.pdf?download=false.
8. Jouyban, A.; Soltanpour, S., Prediction of Dielectric Constants of Binary Solvents at Various Temperatures. *J. Chem. Eng. Data* **2010,** *55*, 2951-2963.
